# Effectiveness of Baricitinib in Refractory Seronegative Rheumatoid Arthritis and Uveitis: A Case Report

**DOI:** 10.3389/fmed.2021.764067

**Published:** 2022-01-14

**Authors:** Yutaka Kaneko, Takanori Murakami, Koichi Nishitsuka, Yuya Takakubo, Michiaki Takagi, Hidetoshi Yamashita

**Affiliations:** ^1^Department of Ophthalmology and Visual Sciences, Yamagata University Faculty of Medicine, Yamagata, Japan; ^2^Department of Ophthalmology, Yamagata Prefectural Central Hospital, Yamagata, Japan; ^3^Department of Orthopaedic Surgery, Yamagata University Faculty of Medicine, Yamagata, Japan; ^4^Yamagata City Healthcare Center, Yamagata, Japan

**Keywords:** JAK inhibitor, baricitinib, uveitis, rheumatoid arthritis, new therapeutic target

## Abstract

Baricitinib is a Janus kinase (JAK) inhibitor used to treat refractory rheumatoid arthritis and blocks the subtypes JAK1 and JAK2. A 35-year-old man with seronegative rheumatoid arthritis complicated by bilateral severe non-granulomatous panuveitis was resistant to steroid treatment, multiple conventional disease-modifying antirheumatic drugs (methotrexate and salazosulfapyridine), and TNF-α inhibitors (adalimumab and infliximab). Therefore, the TNF-α inhibitors were switched to baricitinib to decrease the activity of systemic arthritis. Along with the amelioration of inflammatory activity in seronegative rheumatoid arthritis, the inflammatory activity of uveitis was decreased. Vitreous opacity, serous retinal detachment, and anterior chamber cells showed improvement. Baricitinib was effective not only in refractory systemic arthritis but also in uveitis, which may provide a new treatment option for patients with refractory uveitis.

## Introduction

Rheumatoid arthritis (RA) is a systemic inflammatory disease associated with several extra-articular organ manifestations involving the skin, heart, lungs, and eyes. The pharmacological treatment of RA involves anti-inflammatory analgesics, steroids, conventional synthetic disease-modifying anti-rheumatic drugs (csDMARDs) such as methotrexate, and biological agents, including TNF-α inhibitors (biological DMARDs bDMARDs). Early interventions using these agents have been reported to enable clinical, structural, and functional remission. However, treatment resistance has also been noted with the use of these medications, along with long-term systemic complications caused by administering high doses of steroids and immunosuppressive agents (1). In addition, ocular manifestations of RA, such as dry eye, corneal ulcer, episcleritis, scleritis, and retinal vasculitis, often require the use of topical and systemic steroids as well as immunosuppressive agents (2–4). In recent years, a new class of targeted synthetic DMARDs (tsDMARDs), Janus kinase (JAK) inhibitors, has shown good therapeutic results in such cases (1). Therefore, the aim of this study was to present our experience with treating a patient affected by RA and uveitis using a JAK inhibitor.

## Case Description

**Figure 1:**
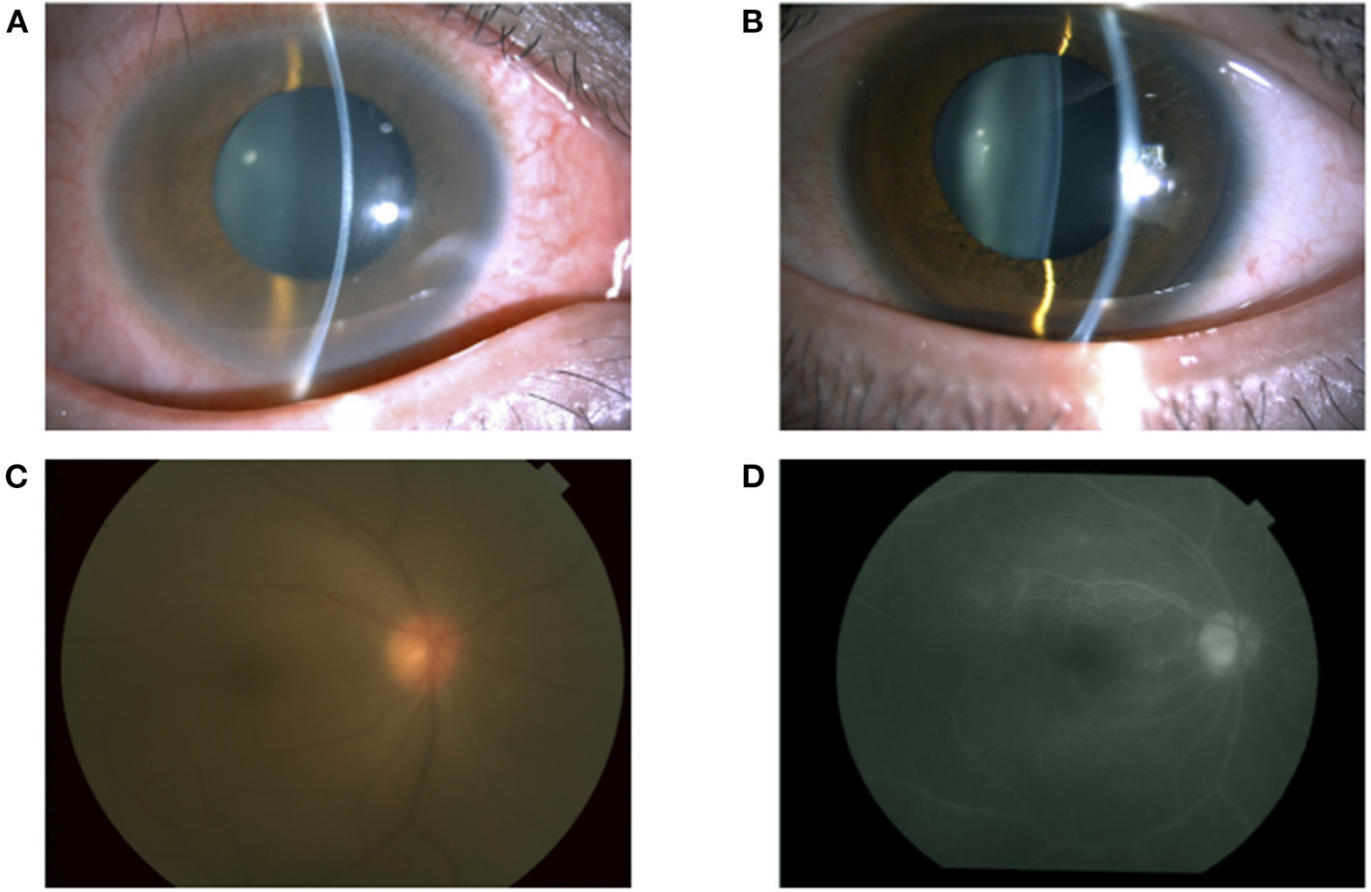
A slit-lamp examination showing conjunctival hyperemia and anterior chamber cells in the right eye **(A)** and left eye **(B)**. A fundus photograph of the right eye showing vitreous opacity **(C)**. Fluorescein angiography of the right eye showing vascular leakage **(D)**.

A 35-year-old man with a medical history of postoperative maxillary osteomyelitis, postoperative vocal cord tumor, right scaphoid fracture, and mediastinal tumor was referred to our hospital. He presented with lower back pain and joint pain in both hands approximately a year ago, as well as the blurred vision of his right eye that he noticed 2 months prior. The postoperative maxillary osteomyelitis was observed 10 years ago and was no longer observed at the initial visit. Vocal cord tumors and mediastinal tumors were biopsied by otolaryngology and thoracic surgery in our hospital, and the pathological result was an inflammatory pseudotumor. At the first visit, his best-corrected visual acuity (BCVA) was 20/16 in both eyes, and the intraocular pressure was 14 mmHg and 10 mmHg in the right and left eyes, respectively. A slit-lamp examination demonstrated conjunctival hyperemia and anterior chamber cells in both eyes (Figures 1A,B). Fundus examination revealed mild vitreous opacity in the right eye (Figure 1C), but no vitreous opacity in the left eye. Optical coherence tomography (OCT) showed no macular edema in either eye. Furthermore, fluorescein angiography revealed retinal vasculitis in the right eye (Figure 1D).

**Figure 2:**
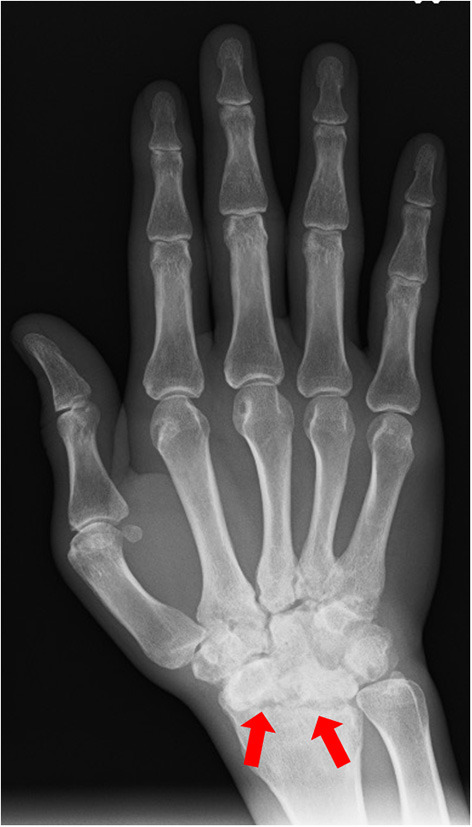
Radiography showing bone erosions of the wrist joint (red arrow) (image of right hand).

He was referred to orthopedic surgery in our hospital due to lower back pain and joint pain in both hands. Radiography showed bone erosions in both wrist joints (Figure 2). Blood tests showed an increase in the white blood cell count (13,530/μL) and C-reactive protein (CRP) levels of 2.37 mg/dL. The rheumatoid factor (RF) and anti-citrullinated protein antibody (ACPA) levels were within normal limits, and human leukocyte antigen B27 (HLA-B27) test was negative. According to the 2010 ACR/EULAR Rheumatoid Arthritis Classification Criteria (5), a score of 7 out of 10 was obtained; the joint distribution was 5 points because he affected over 10 joints, including the bilateral wrist and shoulder joints, metacarpophalangeal (MCP) of the thumb, proximal interphalangeal (PIP) joints of his bilateral index, long, ring, and little finger. Serology was 0 points, symptom duration was 1 point (>6 weeks) and acute phase reactions were 1 point (abnormal CRP). Computed tomography showed no obvious inflammatory findings in the spine and sacroiliac joints, so sacroiliitis was excluded. His final diagnosis was bilateral panuveitis associated with seronegative RA.

**Figure 3:**
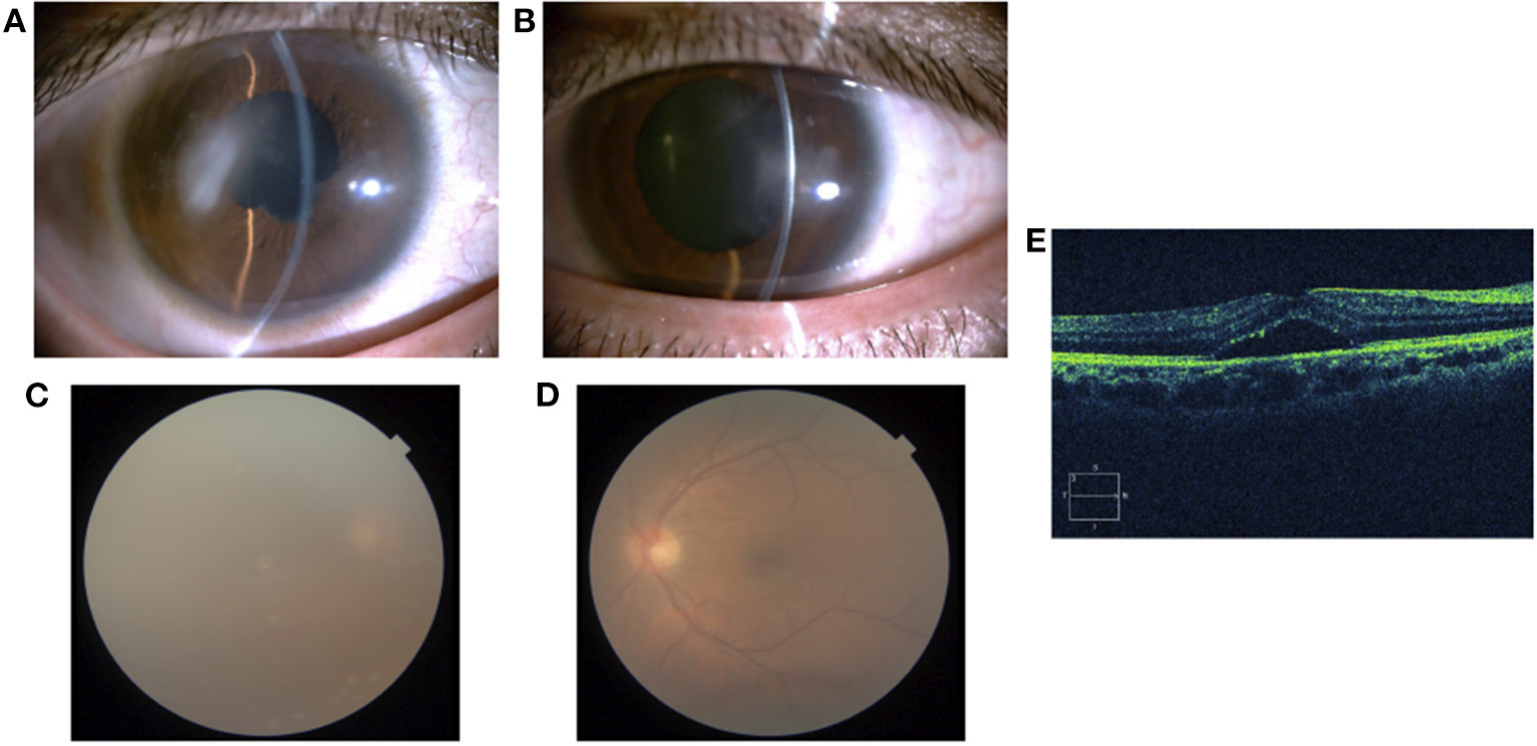
A slit-lamp examination showing corneal infiltration lesion, anterior chamber cells, posterior iris synechia, and anterior subcapsular cataract in the right eye **(A)** and corneal infiltration lesion in the left eye **(B)**. A fundus photograph showing vitreous opacity in the right eye **(C)** and left eye **(D)**. OCT of the right eye showing serous retinal detachment **(E)**.

**Figure 4:**
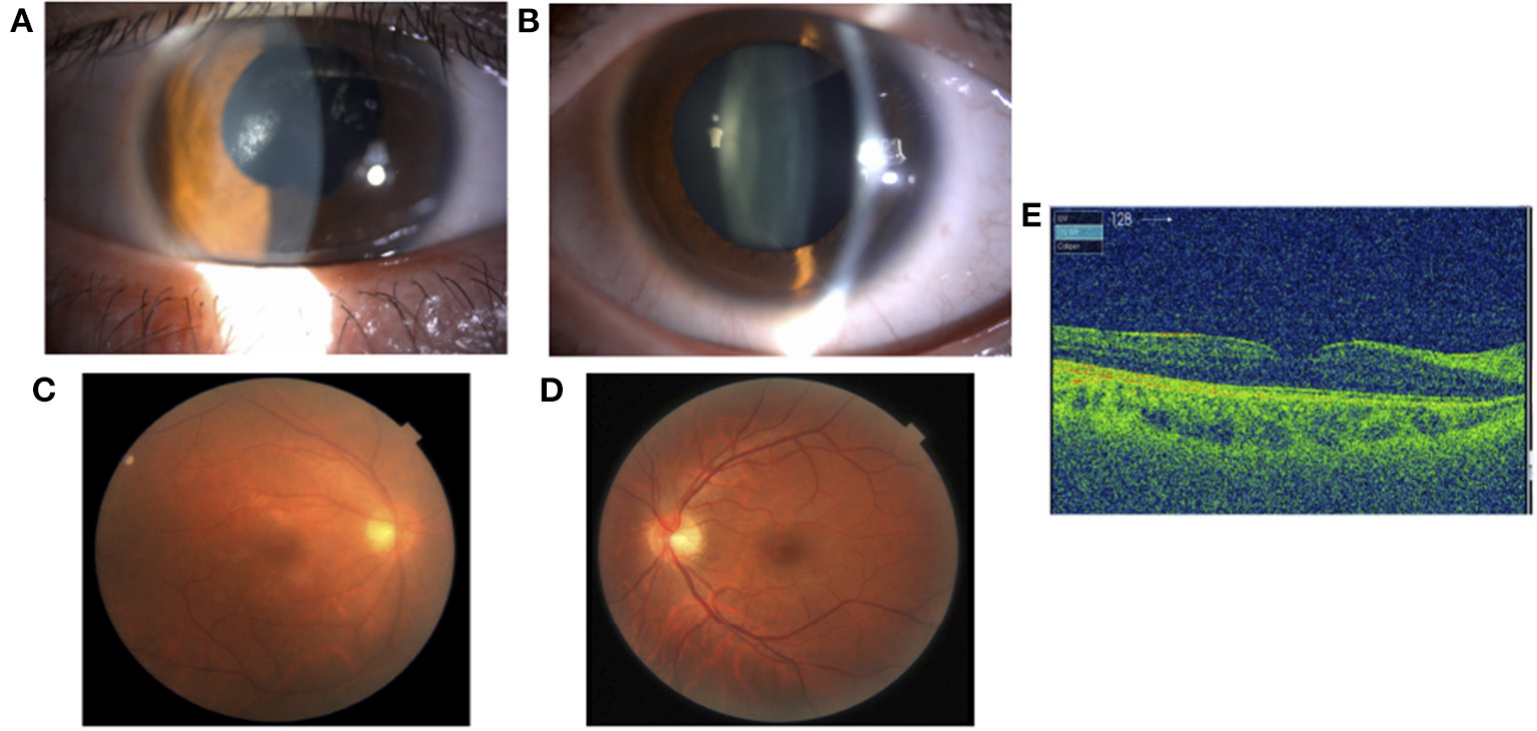
A slit-lamp examination showing improvement of the corneal infiltrative lesions and the anterior chamber cells in the right eye **(A)** and left eye **(B)**. A fundus photograph showing improvement of vitreous opacity in both eyes **(C)** and **(D)**. OCT showing disappearance of serous retinal detachment in the right eye **(E)**.

Treatment with betamethasone ophthalmic solution was started at the first visit, but 2 months later, the vitreous opacity of the right eye worsened and the visual acuity of the same eye decreased to the counting finger; therefore, prednisolone (60 mg/day) was started by the ophthalmologist. After 4 months, systemic arthritis worsened. Consequently, methotrexate (12 mg/week) and salazosulfapyridine (1,000 mg/day) were added to the patient regimen by a rheumatologist. Following this, subcutaneous injection of adalimumab (40 mg/2 weeks) was administered 19 months later due to further exacerbation of arthritis; however, no improvement was observed. Subsequently, the subcutaneous injection of adalimumab was changed to an intravenous infusion of infliximab (3–6 mg/kg) 38 months later. Nevertheless, since neither his systemic arthritis nor uveitis improved after 42 months, the orthopedic surgeon changed the patient's regimen from intravenous infliximab to oral baricitinib (8 mg/day). At the start of the oral baricitinib therapy, CRP levels were as high as 11.03 mg/dL, BCVA was 20/20 and 20/16, and intraocular pressure was 12 mmHg and 11 mmHg in the right and left eyes, respectively. A slit-lamp examination revealed corneal infiltration lesions in both eyes. Furthermore, anterior chamber cells, posterior iris synechia, and anterior subcapsular cataract were observed in the right eye, while no anterior chamber cells were seen in the left eye (Figures 3A,B). Fundus examination revealed vitreous opacity in both eyes (Figures 3C,D), and OCT revealed serous retinal detachment in the right eye (Figure 3E). Three months after the initiation of oral baricitinib, his CRP levels decreased to 0.26 mg/dL, and the corneal infiltrative lesions, as well as the anterior chamber cells, improved (Figures 4A,B). Moreover, systemic arthritis and vitreous opacity in both eyes improved (Figures 4C,D). OCT revealed that serous retinal detachment in the right eye had disappeared (Figure 4E). Two years and seven months after the initiation of baricitinib, his BCVA was 20/20 in both eyes, and no relapse of uveitis was observed. In addition, no serious side effects of oral baricitinib were observed or reported.

## Discussion

RA is classified as seropositive or seronegative based on the presence of RF and ACPA. The incidence of seronegative RA is estimated to be between 16 and 26% of RA patients (6, 7). Seronegative RA has been reported to have lower disease activity and better prognosis than seropositive RA (8). However, Barra et al. reported that seronegative RA showed higher inflammatory activity than seropositive RA, and bone erosion is equivalent over a 2-year course in DMARD-naive patients (6). Therefore, seronegative RA does not appear to be a benign subtype of RA and requires the same intensive treatment as seropositive RA (7). Ocular manifestations of RA include keratoconjunctivitis sicca, episcleritis, scleritis, corneal ulcer, and retinal vasculitis. In particular, corneal ulcer and scleritis are known to become intractable and cause serious visual dysfunction, such as corneal and scleral perforation, macular edema, and secondary glaucoma (2).

This case showed hypopyon, panuveitis, serous retinal detachment, and infiltrative lesions in the center of the cornea, which were not typical ocular complications, as previously reported in rheumatoid arthritis. Therefore, Behcet's disease was suspected, and he was referred to the dermatology in our hospital. However, there were no recurrent oral ulcers, recurrent genital ulcers, or skin lesions. Fluorescein angiography did not show “Fern-like” fluorescein leakage, and Behcet's disease was ruled out. Infiltrative lesions in the center of the cornea were observed simultaneously in both eyes, did not show dendritic epithelial ulcer, and improved after the start of oral baricitinib without the use of antiviral drugs. Viral etiology, such as HSV/VZV, was also ruled out. The incidence of ocular manifestations has been shown to not present any statistically significant difference between patients with seronegative and seropositive RA. The longer the duration of the disease, the larger the number of extra-articular manifestations (9); thus, these ocular manifestations require the same intensive treatment as RA.

JAK is a kinase that is constitutively bound to a cytokine receptor. When a cytokine binds to the receptor, phosphorylation of the transcription factor signal transducer and activator of transcription (STAT) is induced along with phosphorylation of JAK. Phosphorylated STATs form dimers and translocate into the nucleus without the intervention of other signaling molecules to regulate transcription, enabling the regulation of nuclear gene expression by extracellular cytokines. JAK is composed of four molecules, namely JAK1, JAK2, JAK3, and Tyk2, which are activated in different combinations by various cytokines (9).

JAK inhibitors are molecular-targeted synthetic drugs that specifically inhibit JAK. Biological agents, which are high molecular weight proteins, are limited to administration via intravenous or subcutaneous injections. On the other hand, JAK inhibitors are low molecular weight compounds that can be orally administered and are as effective as biological agents. These inhibitors competitively bind to the ATP-binding site of JAK in the cell and specifically inhibit the activity of JAK induced by cytokine stimulation (10, 11). Baricitinib inhibits JAK1 and JAK2, while tofacitinib inhibits JAK1, JAK2, and JAK3. JAK inhibitors such as these are often reported to be effective in RA that is resistant to treatment with biological agents (12–16). However, adverse events such as herpes zoster, serious heart-related events, and cancer are known to occur more frequently following the use of JAK inhibitors when compared to that of biological agents (12, 13).

At present, very little evidence is available on the use of JAK inhibitors for the treatment of ocular inflammation (17–21). Miserocchi et al. reported that baricitinib and tofacitinib showed a significant reduction in inflammatory activity in uveitis than in systemic arthritis when four cases of juvenile idiopathic arthritis-associated uveitis (JIAU) refractory to csDMARD and bDMARD were investigated (17). During their study, no serious systemic side effects required discontinuation of treatment (17).

Bauermann et al. (18) described tofacitinib as a successful strategy for severe refractory uveitis and macular edema; therefore, JAK inhibitors were postulated as a treatment option in select cases that do not respond well to csDMARD and bDMARD or intraocular steroid implants.

Based on the above-mentioned findings, Chen et al. (19) reviewed the current treatment algorithms for JIAU, which are unable to taper local and oral steroids, and recommended treatment with methotrexate followed by TNF-α inhibitors such as adalimumab. IL-6 inhibitors, T cell co-stimulation modulators, JAK inhibitors, and CD20 inhibitors—which have been proven to be effective against arthritis following their recent review for the treatment of JIAU—have been proposed as remedial options when refractory to the above-mentioned treatments (19). In addition, an international, multicenter, open-label controlled study sponsored by Eli Lilly and Company [NCT04088409; 2019–present; (22)] is currently underway to compare baricitinib and adalimumab in JIAU and chronic anterior anti-nuclear antibody positive uveitis. If the effectiveness of baricitinib for these uveitis cases can be proven, it may be effective for other types of uveitis in the future.

In the current case, treatment with csDMARDs or bDMARDs did not suppress the inflammatory activity of systemic arthritis and uveitis. However, after the initiation of oral baricitinib, which is a tsDMARD, inflammatory activity improved not only in systemic arthritis but also in the uveitis of both eyes for a long time. Additionally, good visual acuity was maintained without serious side effects.

Currently, the only approved treatments for uveitis in Japan are steroids, cyclosporine, infliximab, and adalimumab, which have to be administered for a long period in refractory cases. Therefore, considering the systemic and local complications associated with the long-term use of these conventionally prescribed agents, new therapeutic agents with different mechanisms of action are desired. In this report, we highlight the efficacy and safety of baricitinib for the treatment of uveitis with RA resistant to conventional treatment. In the future, the use of a JAK inhibitor along with the involvement of a multidisciplinary team including the rheumatologist may be a viable option if the treatment by an ophthalmologist is not sufficient in similar cases.

## Data Availability Statement

The original contributions presented in the study are included in the article/supplementary material, further inquiries can be directed to the corresponding author/s.

## Ethics Statement

The studies involving human participants were reviewed and approved by the Ethical Review Committee of Yamagata University Faculty of Medicine. The patients/participants provided their written informed consent to participate in this study. Written informed consent was obtained from the individual(s) for the publication of any potentially identifiable images or data included in this article.

## Author Contributions

YK: concept and design of study or analysis and interpretation of data. YK and YT: acquisition of data. TM, KN, YT, MT, and HY: drafting the article or revising it critically for important intellectual content and final approval of the version to be published. All authors contributed to the article and approved the submitted version.

## Conflict of Interest

The authors declare that the research was conducted in the absence of any commercial or financial relationships that could be construed as a potential conflict of interest.

## Publisher's Note

All claims expressed in this article are solely those of the authors and do not necessarily represent those of their affiliated organizations, or those of the publisher, the editors and the reviewers. Any product that may be evaluated in this article, or claim that may be made by its manufacturer, is not guaranteed or endorsed by the publisher.
